# Serum Free Zinc Is Associated With Vaccination Response to SARS-CoV-2

**DOI:** 10.3389/fimmu.2022.906551

**Published:** 2022-06-30

**Authors:** Thilo Samson Chillon, Maria Maares, Kamil Demircan, Julian Hackler, Qian Sun, Raban A. Heller, Joachim Diegmann, Manuel Bachmann, Arash Moghaddam, Hajo Haase, Lutz Schomburg

**Affiliations:** ^1^Institute for Experimental Endocrinology, Berlin Institute of Health, Charité-Universitätsmedizin Berlin, Corporate Member of Freie Universität Berlin, Humboldt-Universität zu Berlin, Berlin, Germany; ^2^Department of Food Chemistry and Toxicology, Technische Universität Berlin, Berlin, Germany; ^3^Bundeswehr Hospital Berlin, Clinic of Traumatology and Orthopaedics, Berlin, Germany; ^4^Department of General Practice and Health Services Research, Heidelberg University Hospital, Heidelberg, Germany; ^5^Aschaffenburg Trauma and Orthopaedic Research Group (ATORG), Center for Orthopaedics, Trauma Surgery and Sports Medicine, Hospital Aschaffenburg-Alzenau, Aschaffenburg, Germany; ^6^Orthopedic and Trauma Surgery, Aschaffenburg, Germany

**Keywords:** COVID-19, trace element, antibody, immunoglobulin, vaccine, diagnostics, free zinc, COVID-19 vaccination

## Abstract

**Background:**

Zinc (Zn) is an essential trace element with high relevance for the immune system, and its deficiency is associated with elevated infection risk and severe disease course. The association of Zn status with the immune response to SARS-CoV-2 vaccination is unknown.

**Methods:**

A cohort of adult health care workers (n=126) received two doses of BNT162B2, and provided up to four serum samples over a time course of 6 months. Total SARS-CoV-2 IgG and neutralizing antibody potency was determined, along with total as well as free Zn concentrations.

**Results:**

The SARS-CoV-2 antibodies showed the expected rise in response to vaccination, and decreased toward the last sampling point, with highest levels measured three weeks after the second dose. Total serum Zn concentrations were relatively stable over time, and showed no significant association with SARS-CoV-2 antibodies. Baseline total serum Zn concentration and supplemental intake of Zn were both unrelated to the antibody response to SARS-CoV-2 vaccination. Time resolved analysis of free Zn indicated a similar dynamic as the humoral response. A positive correlation was observed between free Zn concentrations and both the induced antibodies and neutralizing antibody potency.

**Conclusion:**

While the biomarkers of Zn status and supplemental Zn intake appeared unrelated to the humoral immune response to SARS-CoV-2 vaccination, the observed correlation of free Zn to the induced antibodies indicates a diagnostic value of this novel biomarker for the immune system.

**Graphical Abstract:**
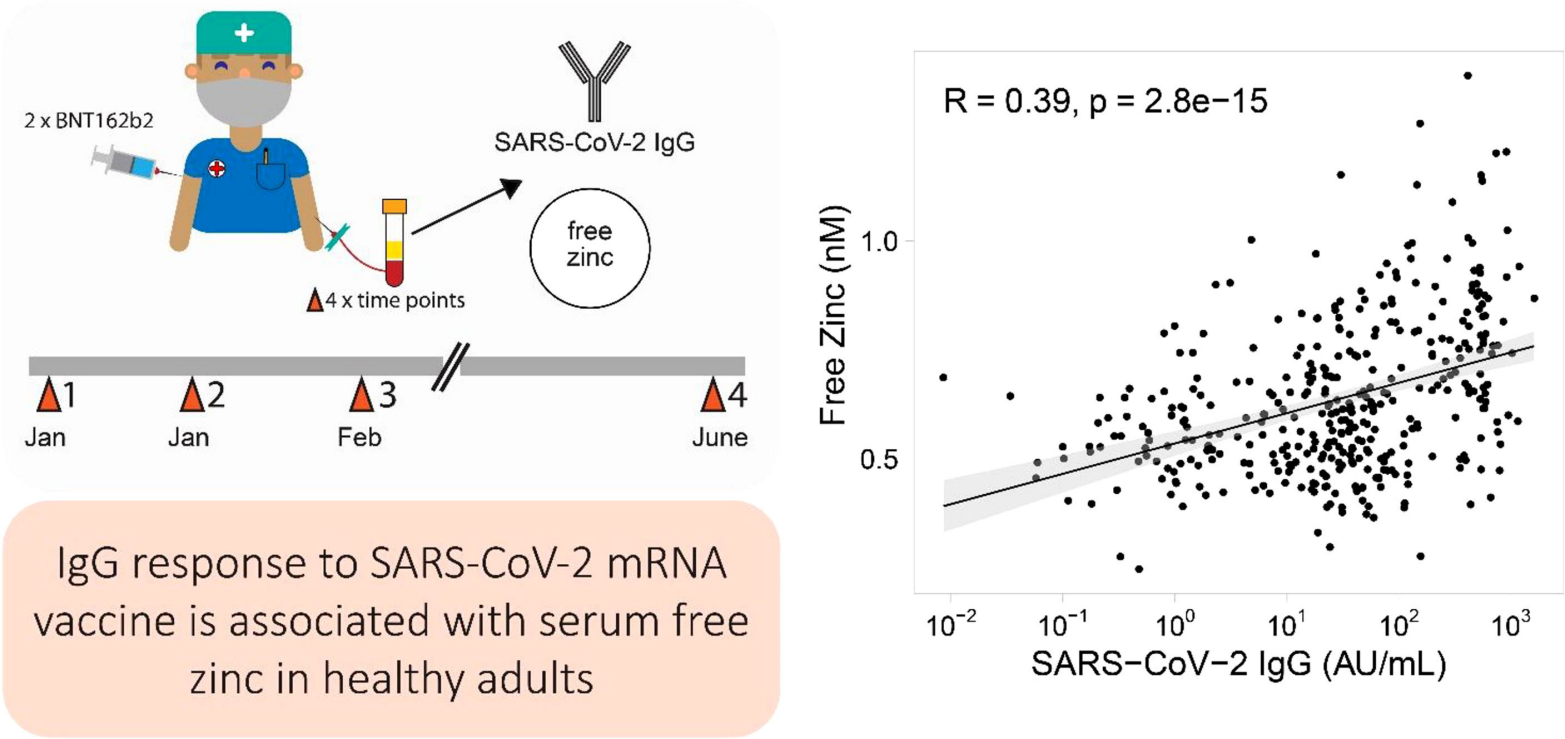


## Introduction

The essential trace element zinc (Zn) is required as cofactor for the structure and function of a high number of proteins and enzymes (1, 2). It also participates in signaling pathways and supports or even mimics the functions of hormones, growth factors, and cytokines (3). Moreover, it contributes essentially to many aspects of innate and adaptive immunity (4–9), including the maturation of dendritic cells, mast cell activation, and T cell maturation (6, 8, 10). Zinc supplementation has been shown to promote a Th1 response (11), whereas Zn deficiency is associated with cell-mediated immune dysfunction due to a shift from Th1 to Th2 (6). In addition, pronounced Zn deficiency impairs production of cytokines such as interferon-gamma, interleukin-2 (IL-2), and tumor necrosis factor-alpha (TNF-α) (12, 13).

Intracellular Zn has been described as a potent second messenger/signaling ion (14–16), which may be released in response to extracellular signals, e.g. in mast cells upon stimulation of the high-affinity IgE receptor, resulting in a phenomenon called a zinc wave (17). Hereby, MAPK activity and expression of interleukin-6 and TNF-α genes are regulated *via* Zn-dependent inhibition of phosphatase activity (14, 16). Similarly, stimulation of T-cell receptors (TCR) leads to an increase in cytoplasmic Zn *via* influx from extracellular sources, enhancing proximal TCR signaling and affecting the T-cell response to antigens and vaccines (15). A strong interrelationship of Zn status with survival odds and mortality risk of patients affected by the ongoing Coronavirus Disease-2019 (COVID-19) pandemic has been described in recent observational studies (18–23). This finding was also supported by *in vitro* studies assessing the effects of Zn2^+^ on SARS-CoV replication and RNA polymerase activity (24). In addition, a potential direct interrelation of Zn was also proposed for SARS-CoV-2 maturation (25, 26). The published studies on immune responses to anti-viral vaccines in relation to Zn status are controversial and inconclusive at present (27, 28). However, in view of the consistently reported interrelationship between serum Zn deficiency and severe COVID-19 course and mortality, we hypothesized that the vaccination response to SARS-CoV-2 immunization correlates with zinc status, in particular with the fraction of free zinc as a potential biomarker of extracellular Zn availability.

## Material and Methods

### Study Design and Study Cohort

All blood samples were collected from healthy adults participating in the observational ATORG Study (29, 30). The authorities in Bavaria, Germany, provided ethical counselling (Ethik-Kommission der Bayerischen Landesärztekammer, Munich, Germany, EA No. #20033), and the study had been registered at the German Clinical Trial Register (Deutsches Register Klinischer Studien, ID: DRKS00022294, Sept. 14th 2020). An amendment to the protocol was added Jan. 12th, 2021 (Ethik-Kommission der Bayerischen Landesärztekammer). All participants enrolled into the observational study provided written informed consent at study entry. The final cohort of subjects analysed consisted of adult male and female health care workers (n=126 at baseline), who received two doses of the Biontech/Pfizer vaccine BNT162b2 during a highly coordinated hospital-wide vaccination process. Serum samples were taken at four consecutive time points, i.e., at first dose of vaccination, at the time of second dose (n= 115) (week three), at week six (n=113) and at week 24 (n=56). The samples obtained were shipped on dry ice to the analytical laboratory in Berlin, Germany, and analysed by scientists and technicians blinded to the clinical data, essentially as described previously (29, 30). All analyses were conducted at Charité-Universitätsmedizin Berlin, except for the analyses of free zinc, which was measured at Technische Universität Berlin.

### Measurement of Antibodies to SARS-CoV-2 and Their Neutralizing Activity

The measurements of antibodies to SARS-CoV-2 IgG and the assessment of neutralizing activity were described earlier (29, 30). In brief, IgG concentrations to SARS-COV-2 were determined with an automated chemiluminescent two-step capture immunoassay (TGS COVID-19, product code: CVCL100G, Immunodiagnostic Systems (ids) Holdings PLC, Frankfurt, Germany). The neutralizing activity was determined *via* a competitive immune-enzymatic colorimetric method, as described (SPIA, Spike Protein Inhibition Assay, product code: DKO205/RUO, ids Holdings PLC) (29, 30).

### Quantification of Total Serum Zn

The concentration of total serum Zn was determined by total reflection X-ray fluorescence (TXRF) using a benchtop TXRF spectrometer (S4 T-STAR, Bruker Nano GmbH, Berlin, Germany), as described (21, 31, 32). Briefly, serum samples were spiked with a gallium standard, applied to polished glass slides and dried. Fluorescence from X-ray activation was recorded by a benchtop TXRF spectrometer and used to calculate trace element concentrations from the emission spectrum.

### Free Zinc Measurement and Ratio of Free to Total Zinc

The concentration of free Zn was determined by a fluorimetric method using the low molecular weight Zn sensor Zinpyr-1 (Santa Cruz biotechnology, Dallas, USA), as described (23, 33, 34). Briefly, 20 μL of serum sample, which had been pre-diluted in assay buffer (1:10) and stored at -80°C, was added to 80 μL pre-warmed assay buffer containing a final concentration 0.05 μM Zinpyr-1. The fractional saturation of the sensor was determined with 15 µL EDTA (800 µM) or 15 µL ZnSO_4_ (4.5 mM) to induce a minimal and maximal fluorescence signal of Zinpyr-1, respectively. Free serum Zn concentrations were calculated using the dissociation constant (Kd) for the Zinpyr-1-Zn-complex of 0.7 nM (35, 36). The ratio of free Zn/total Zn per sample was calculated using the following formula:


$$\frac{\left(Free\;zinc\;\left(nmol/L\right)\;*\;65.38\;g/mol\right)\;*10^{2}}{Total\;Zinc\;\left(\frac{µg}{L}\right)}$$

### Statistical Analysis

Distribution of numerical variables was investigated visually by histogram plots as well as statistically by applying the Shapiro-Wilk-Test (37). When describing baseline patient characteristics, continuous variables were expressed as median (interquartile range (IQR)). Pairwise comparisons were conducted by applying the Wilcoxon-Rank-sum test to detect differences in serum markers between different sampling time points. In addition, time resolved distributions of total serum Zn, free Zn and the free Zn/total serum Zn ratio over time were visualized with ridge-density plots. Spearman’s rank correlation was used to detect correlations between continuous variables. Zinc biomarkers at baseline were categorized into tertiles. Differences in antibody concentrations to SARS-CoV-2 and neutralizing potency across different tertiles of Zn biomarkers were determined by applying the Kruskal-Wallis-test. All statistical analyses were two-sided, and p-values below 0.05 were classified as statistically significant. The statistical analyses were performed using the R software, version 4.1.1, implementing the packages dplyr, tidyr, gtsummary, ggplot2, and ggpubr (38–40).

## Results

### SARS-CoV-2 Antibody Concentrations and Zn Status Over Time

**Figure 1:**
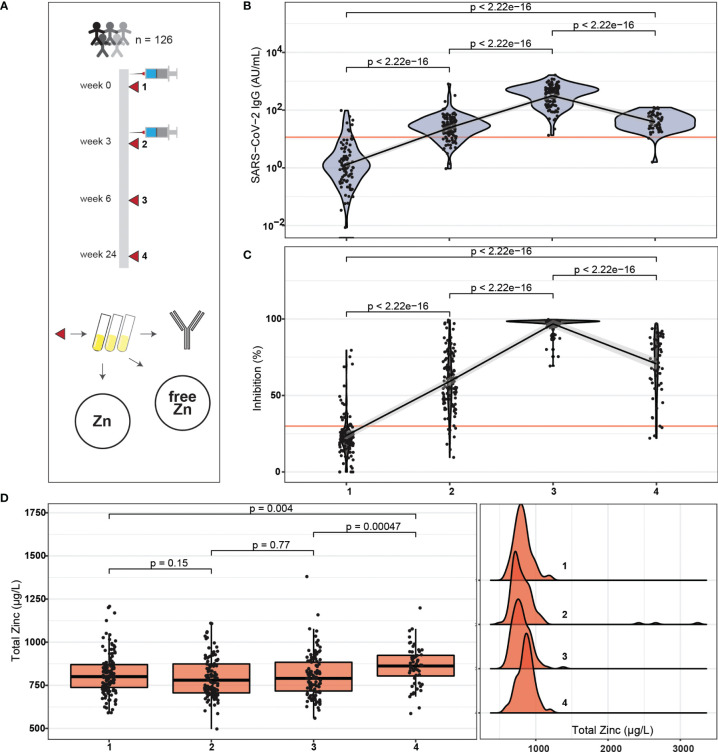
Study design, SARS-CoV-2 antibody response and Zn status during the period of observation. **(A)** Healthy adult subjects received two doses of BNT162b2 vaccine within three weeks. Blood samples were collected on the days of vaccination, as well as three weeks and 21 weeks after second vaccination. The serum samples were analysed for SARS-CoV-2 antibodies, neutralizing activity, and total serum Zn as well as free Zn concentrations. **(B)** Total SARS-CoV-2 antibody concentrations increased from first vaccination to a transient peak at three weeks after second vaccination, and declined thereafter. After six weeks, all participants reached seropositivity. **(C)** The serum samples showed neutralizing activities in parallel to SARS-COV-2 antibody concentrations, again with a transient peak at time point three, and with all participants passing the predefined threshold of positivity (dark orange line). **(D)** Total Zn concentrations were similar during the first three sampling points, but were slightly elevated at study end. All data points are shown including three samples with exceptionally high total serum Zn concentrations (**D**, right hand panel). The data on SARS-CoV-2 antibodies **(B, C)** were presented earlier in relation to vitamin D and Se, and are provided here for orientation. Pairwise comparisons were conducted applying the Wilcoxon-Rank-Sum test.

Healthy adult male and female health care workers (n = 126) were successfully enrolled into this prospective observational study. All participants received two doses of an mRNA-based vaccine (Biontech BNT162b2) within a time frame of three weeks (**Figure 1A**). Four consecutive time points of blood sampling were offered per subject, i. e., on the days of the first and second vaccination, as well as six and 24 weeks after the first vaccination. Most of the subjects contributed a sample at the sampling points one to three, and about one half also provided a sample at time point four, i.e., after 24 weeks. The majority of participants at study start were female (83.3%), and 30% reported self-administered Zn supplementation (**Table S1**). A total of 410 blood samples were finally available for analysis and assessed for SARS-CoV-2 antibody concentrations, neutralizing antibodies inhibiting spike protein binding to ACE2, as well as total and free Zn concentrations, i.e., an average of 3.25 samples/participant. The SARS-CoV-2 antibody levels displayed some variation in the first sample analysed, likely due to previous exposure to the virus and to patients with COVID-19. Non-detectable IgG levels were observed in 35 samples at first time point. At sampling point three, all samples were SARS-CoV-2 antibody positive (>11.5 AU/mL) according to the threshold predefined by the manufacturer. A decrease in SARS-CoV-2 IgG concentrations is seen towards the fourth sampling point (**Figure 1B**). The neutralizing activity of the antibodies showed very similar dynamics to the concentration of antibodies to SARS-CoV-2 across the study (**Figure 1C**). Concentrations of total serum Zn were not different in the first three samples, but appeared slightly elevated in the last sample (**Figure 1D**).

### Alterations in Total Serum Zn and Free Zn Concentrations After Vaccination

**Figure 2:**
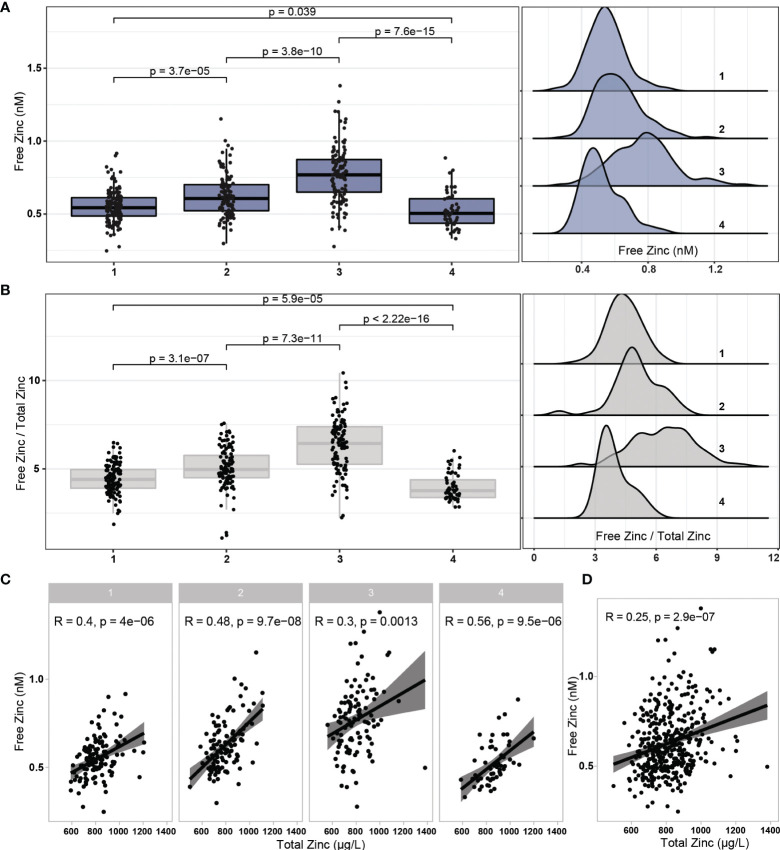
Changes in free Zn concentrations and free Zn/total Zn ratio during the study. **(A)** Free Zn concentrations displayed a transient peak at time point three (six weeks after first vaccination), and a relative minimum at study termination (24 weeks after first vaccination). **(B)** The ratio of free Zn/total serum Zn showed a similar and more pronounced pattern of changes. **(C)** Total serum Zn and free Zn correlated positively at all sampling time points. **(D)** In the full cohort of samples, the parameters free Zn and total serum Zn showed a positive linear correlation. Three data points of exceptionally high serum Zn are not shown due to reasons of scale. Pairwise comparisons were conducted by applying the Wilcoxon-Rank-Sum test. Correlations were analysed by Spearman’s rank correlation.

Concentrations of total serum Zn and free Zn were determined in all samples available, analysed and compared. Free Zn showed a transient increase after vaccination during the period of observation, with a relative peak at the third sampling point, and declining towards a relative minimum at the end of the study (**Figure 2A**). Meanwhile, serum Zn stayed stable over the first three sampling time points, indicating an initial shift from protein bound Zn to unbound free Zn, which was apparently reversed in the last time point. The ratio of free Zn to total serum Zn showed a similar picture, with the differences between the time points being more pronounced (**Figure 2B**). Total serum Zn and free Zn concentrations correlated at all sampling times, with least stringency at the transient peak of free Zn at time point three (**Figure 2C**). A moderate positive correlation in the full cohort of samples analysed was observed (Spearman, R = 0.25, p < 0.001) (**Figure 2D**).

### Vaccination Response in Relation to Baseline Zn Status

**Figure 3:**
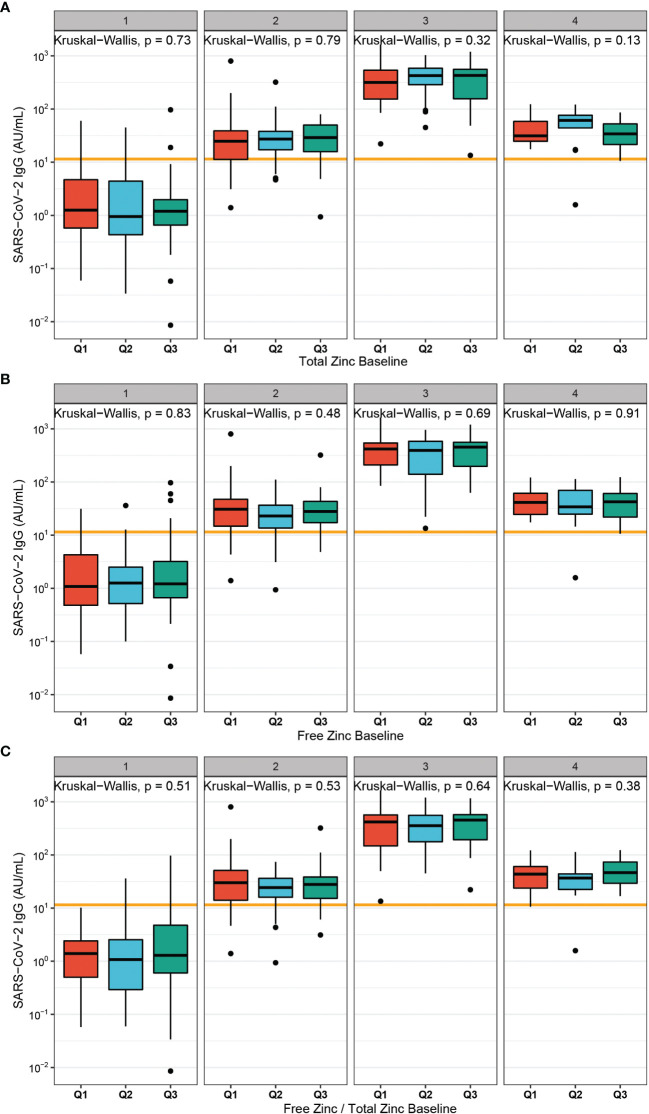
Baseline Zn status in relation to antibodies to SARS-CoV-2 after vaccination. **(A)** Total serum Zn was categorized into tertiles at time of first vaccination (Q1 < 764.3 µg/L; Q2 < 852.4 µg/L; Q3 > 852.4 µg/L), and plotted against SARS-CoV-2 antibody concentrations. No significant differences were observed between the groups. **(B)** Free Zn was divided into tertiles at first vaccination (Q1 < 0.51 nM; Q2 < 0.59 nM; Q3 > 0.59 nM). Again, no significant differences in humoral vaccination response over time was detected. **(C)** The free Zn/total serum Zn ratio was calculated and used to classify the samples into tertiles (Q1 < 4.09; Q2 < 4.81; Q3 > 4.81). No significant difference in the concentrations of antibodies to SARS-CoV-2 were detected across the tertiles. Two-sided Kruskal-Wallis test was used to assess differences.

Initial Zn status of the subjects at first vaccination was separated into tertiles (Q1-Q3). A comparison of antibody levels to SARS-CoV-2 developing after vaccination according to total serum Zn (**Figure 3A**), free Zn (**Figure 3B**), or the free Zn/total serum Zn ratio (**Figure 3C**) indicated no significant differences. The same result was obtained for initial Zn status and neutralizing activity of the samples (**Figure S1**).

### Effects of Self-Administered Zn Supplementation on the Vaccination Response

**Figure 4:**
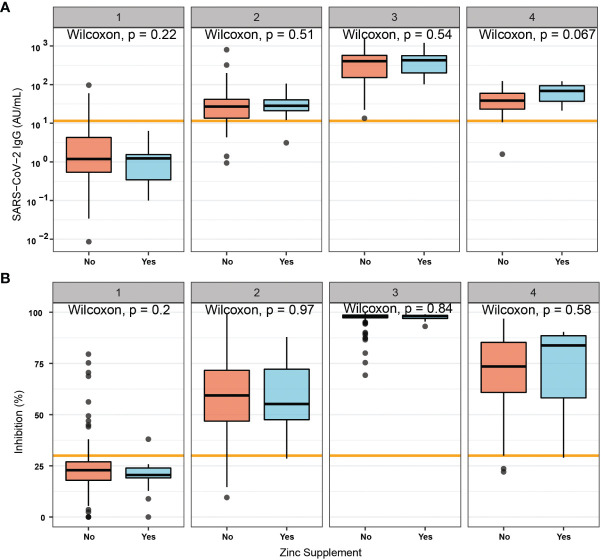
Vaccination response in relation to self-administered supplemental Zn intake. A subset of probands reported no (No) or active (Yes) self-administered intake of Zn-containing supplements during the study. Immune responses measured by **(A)** total antibody concentrations to SARS-CoV-2, or by assessment of **(B)** binding inhibition were not significantly different between the two groups. Pairwise comparisons were conducted by applying the Wilcoxon-Rank-Sum test.

A subgroup of the study probands reported self-administered intake of Zn-containing supplements before and/or during the time of vaccination (**Table S1**). The concentrations of total serum Zn (**Figure S2A**) and free Zn (**Figure S2B**) were not significantly different in relation to supplemental intake. The analysis of antibodies induced in response to vaccination with respect to supplemental Zn intake showed no significant differences between the two groups of subjects classified according to reported self-administered Zn supplementation (**Figure 4**).

### Relationship of Zn Parameters With Antibodies to SARS-CoV-2

**Figure 5:**
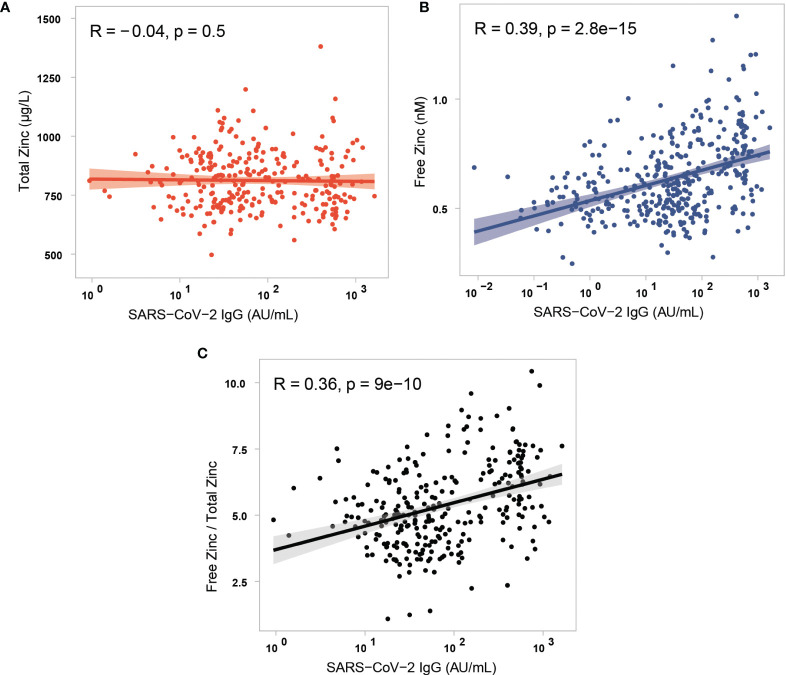
Correlation analysis of the three different parameters of serum Zn status with antibodies to SARS-CoV-2. **(A)** Total serum Zn concentrations and antibodies to SARS-CoV-2 showed no significant correlation in the study cohort (three data points of very high serum Zn are not shown in the figure for reasons of scale). The parameters **(B)** free Zn concentrations, and **(C)** free Zn/total Zn ratio correlated positively and significantly with antibodies to SARS-CoV-2. Data were analysed by Spearman’s rank correlation.

The three parameters of Zn status, i.e., total serum Zn, free Zn and the free Zn/total serum Zn ratio were compared to the induced antibodies to SARS-CoV-2. There was no significant correlation between total serum Zn and SARS-CoV-2 antibody concentrations (**Figure 5A**). In comparison, free Zn concentrations correlated with antibodies to SARS-CoV-2 (**Figure 5B**). Accordingly, the free Zn/total serum Zn ratio showed a similar strong correlation to the concentration of SARS-CoV-2 antibodies (**Figure 5C**). A parallel analysis was conducted for the interrelationship between the Zn parameters and the neutralization activity of the serum samples. Again, total serum Zn was not correlated to antibody-mediated neutralizing activity (**Figure S3A**), whereas free Zn and the free Zn/total serum Zn ratio showed a significant positive correlation to inhibition activity of the induced SARS-CoV-2 antibodies (**Figures S3B, C**).

## Discussion

This study reports an analysis of three parameters of serum Zn status in relation to the response to SARS-CoV-2 vaccination in healthy adult subjects over the course of 24 weeks. Total serum Zn concentrations showed no significant association with the vaccination response, but free Zn and a composite biomarker represented by the ratio of free Zn/total serum Zn concentrations correlated stringently to the induced antibodies. Qualitatively, the same results were obtained when comparing the Zn biomarkers to the neutralizing activity of the antibodies, measured as the disrupting effect on the binding of recombinant spike protein to the SARS-CoV-2 receptor ACE2; again, free Zn or the ratio of free Zn/total serum Zn showed significant positive correlations to neutralizing activity, whereas there was no interrelationship with total serum Zn concentrations. Collectively, our data indicate that the small fraction of labile-, or non-protein-bound Zn ions, which is denoted as free Zn, constitutes a promising biomarker of Zn status for clinical research. The positive correlation of free Zn concentrations with the vaccination response may indicate a general beneficial interrelationship of free Zn with the activity of the immune system, with potential relevance also in other conditions, e.g., in infectious, inflammatory or autoimmune diseases, albeit this hypothesis needs to be tested in future analyses.

Under regular conditions, the physiological amount of Zn in serum corresponds to ca. 1% of total body Zn. Serum levels in heathy humans range from 750 to 920 µg/L (11.5- 15.0 µM) (41), with Zn deficiency according to the 2.5^th^ percentile of healthy European subjects starting at as low as 642.5 μg/L (21, 42). In human serum, the essential trace element is mainly transported bound to albumin (60%), α-macroglobulin (30%) and transferrin (10%) (43). It can be assumed that due to the replete Zn status of the enrolled participants of the study, the self-reported supplementation was not yielding increased serum Zn concentrations. However, it can not be excluded that self-supplementation caused increased intracellular Zn levels, which is not necessarily reflected in serum concentrations. For this reason, a lack of effect on circulating Zn concentration does not exclude a modulatory effect on Zn-dependent biochemical pathways in certain target cells. In addition to protein-bound Zn, several studies have indicated the presence of a small fraction of free, labile- or non-protein-bound Zn ions (44), which may interact with low molecular weight ligands and thereby constitute the available and biologically active Zn species in human blood. There are indications that this fraction is responsible for cellular activities, enzymatic functions and internalization, as convincingly shown in cell based studies and preclinical models (23, 34, 45). The assessment of this fraction is considered as a useful biomarker of Zn status (46), potentially reflecting physiologically relevant alterations in Zn availability and homeostatic adaptations to certain challenging conditions (2). However, clinical studies comparing the different fractions of protein-bound versus free Zn ions are very few, and no significant differences in relation to clinical parameters have been reported so far. To our knowledge, the present study is the first investigation of a potential interrelationship between the humoral immune response and free zinc in human subjects.

For this reason, the present study was conducted, hypothesizing that total serum Zn and free Zn both being of importance for successful vaccination response. Only the fraction of free Zn ions showed a significant correlation to the vaccination-induced increase in SARS-CoV-2 antibodies. Until now, the available observational data on an interrelationship of Zn with the antibody response to an infection by SARS-CoV-2 have been rather controversial (47, 48), potentially due to unknown confounding factors.

Several clinical studies assessing serum Zn in patients with COVID-19 reported consistently that low serum Zn concentrations are associated with severe disease, poor clinical outcome, and death risk (20, 21, 49, 50). COVID-19 patients with Zn deficiency displayed a higher rate of complications, higher odds of acute respiratory distress syndrome (20), and longer time span until recovery (20, 50). Furthermore, a sufficiently high Zn status appeared essential to support the therapeutic measures in COVID-19 treatment (51–53). Targeted intervention has indicated that supplemental Zn positively affects lymphocyte counts as compared to untreated patients (51). Fast treatment of newly infected subjects with a combination of supplemental Zn, low-dose hydroxychloroquine and azithromycin successfully reduced hospitalization rate (52). Longer times of active Zn supplementation proved efficient to protect from SARS-CoV-2 infection risk and severe COVID-19 course (54). These results were in line with prior analyses on a protective effect of supplemental Zn on development of common colds (55). An intravenous application would be an alternative and fast measure to correct and reverse Zn deficiency in the acute phase of disease (53, 56). However, some intervention studies also showed no supplementation effects, e.g. no benefit was observed in the randomized placebo-controlled COVID A to Z trial (53). Similarly, Zn supplementation in the elderly was inefficient in improving antibody response after influenza (57) or hepatitis B vaccination (58). Collectively, the current picture highlights several positive associations of Zn with an efficient function of the immune system in the defense towards infection or the mounting antibody response upon vaccination; yet some intervention studies were unsuccessful, showing null results, and others reported ambiguous findings (19, 56, 59, 60). In this respect, an analysis of the fraction of free Zn and its comparison to total serum Zn concentrations may be helpful to providing a better and more consistent picture on the role of Zn in infectious diseases and upon vaccination, and for identifying the confounders affecting the relative proportion of both biomarkers of Zn status. The time resolved analysis of the immune biomarkers, of serum Zn and free Zn also revealed a pattern indicating an initial shift of bound Zn to free Zn after vaccination, which is apparently reversed 21 weeks after the second vaccination. This shift in the ratio of bound to free Zn may constitute a consequence of vaccination and concomitant inflammation, yet a longer time period is needed to assess how the reversion of the initial shift behaves in long term, and whether this displays a SARS-CoV-2 vaccine-specific finding or a more general mechanism. Future investigation into whether this initial shift is a general response and applies similarly to other vaccines or inflammatory states is particularly important, as free Zn constitutes the biologically active component of serum Zn. While it is needed for various beneficial purposes and might be taken up by cells in need more readily and efficiently, free Zn is also associated with toxic properties (45), which may become relevant upon sudden increase and as a direct response to inflammation or vaccination (61, 62).

Among the strengths of our analysis are the well-designed vaccination program underlying our observational study, and the standardized collection, transport and measurement of the serum samples at a remote location by experienced scientists blinded to any clinical information. The parallel quantification of two biomarkers of vaccination response and of two Zn-dependent serum parameters giving rise to three complementary biomarkers of Zn status constitutes another strength. Finally, the clear-cut results falsifying our initial hypothesis on the prime importance of total serum Zn concentrations, but highlighting the potential value of free Zn concentrations as a physiologically relevant immune-related parameter of Zn status, further support the value of this study.

Among the limitations are the focus on humoral immune response only, i.e., the measurement of circulating antibodies, without an assessment of cell-based immune responses. Moreover, the nature of our analysis as an observational study precludes conclusions on mechanisms and causality, and there is a general lack of molecular understanding that might give rise to the observed differences between total serum Zn and free Zn concentrations in relation to the immune response. Additionally, the time points of analysis were few, and a more frequent sampling scheme would have provided a more detailed picture on the dynamic alterations occurring in response to vaccination. And finally, a larger study cohort would have allowed for a better statistical analysis taking additional confounders such as dietary patterns, additional anthropometric differences, and other acute parameters of health and disease into account. Further, our study did not include any random sampling method for enrolment, which may limit the generalizability of the results. In addition, our study considered healthy adults only with a moderate baseline Zn status; seniors, children, adolescents or diseased patients were not included, and our data cannot be extrapolated to these groups of subjects.

Nevertheless, in view of the general correlation between total serum Zn and free Zn and at the same time the strong differences in correlation strengths to the induced antibodies, we consider the herein provided findings as highly relevant for the clinical assessment of Zn status, as they highlight a potential value of free Zn as a novel promising biomarker associated with vaccination and humoral immune response. Therapeutic measures to positively modify the fraction of free Zn and the identification of disruptors of free Zn mobilisation may enable further insights into the endogenous pathways regulating Zn homeostasis and might provide new clues for adjuvant immunomodulation.

## Data Availability Statement

Anonymised data is available upon reasonable request from the corresponding authors.

## Ethics Statement

The studies involving human participants were reviewed and approved by Ethik-Kommission der Bayerischen Landesärztekammer, Munich, Germany, EA No. #20033. The patients/participants provided their written informed consent to participate in this study.

## Author Contributions

Conceptualization, KD,TC, AM, and LS; methodology, TC, KD, MM, and QS; validation, TC, KD, and LS; formal analysis, TC, KD, MM, and LS; resources, JD, MB, AM, HH, and LS; data curation, TC, KD, MM, and LS; writing—original draft preparation, TC, KD, MM, HH, and LS; writing—review and editing, AM, RH, JD, JH, QS, and MB; software, KD and TC; visualization, KD and TC; supervision, AM and LS; funding acquisition, HH and LS. All authors have read and agreed to the published version of the manuscript.

## Funding

The research and analyses are supported by the Deutsche Forschungsgemeinschaft (DFG), Research Unit FOR-2558 “TraceAge” (Scho 849/6-2, HA 4318/4-2) and CRC/TR 296 “Local control of TH action” (LocoTact, P17), and by the German Federal Ministry for Economic Affairs and Energy (BMWi, ZIM program, project #KK5051601BM0 to LS).

## Conflict of Interest

The authors declare that the research was conducted in the absence of any commercial or financial relationships that could be construed as a potential conflict of interest.

## Publisher’s Note

All claims expressed in this article are solely those of the authors and do not necessarily represent those of their affiliated organizations, or those of the publisher, the editors and the reviewers. Any product that may be evaluated in this article, or claim that may be made by its manufacturer, is not guaranteed or endorsed by the publisher.
