# Supplementary material for: Serum Free Zinc Is Associated With Vaccination Response to SARS-CoV-2
Source: Front Immunol. 2022 Jun 30;13:906551. doi: 10.3389/fimmu.2022.906551 (PMC9280661; doi:10.3389/fimmu.2022.906551)
Supplement: Supplementary file 1 [file DataSheet_1.pdf]

Supplementary Material Serum Free zinc and COVID-19 vaccination

1 Supplementary Figures and Tables

Table S1. Baseline characteristics of study participants stratified by sex

Characteristic	**Overall**, N = 126	**Female**, N = 110	**Male**, N = 16
**Age**	47 (37, 55)	47 (37, 55)	42 (36, 53)
**SARS-CoV-2 IgG** (AU/mL)	0.6 (0.0, 2.0)	0.6 (0.0, 2.0)	0.6 (0.0, 2.5)
**Inhibition** (%)	23 (18, 26)	23 (18, 26)	22 (14, 25)
**Total Zn** (µg/L)	800 (738, 870)	798 (737, 869)	837 (754, 938)
**Free Zn** (nM)	0.54 (0.49, 0.61)	0.54 (0.49, 0.62)	0.52 (0.48, 0.58)
**Free Zn/ Total Zn**	4.40 (3.91, 4.96)	4.48 (3.97, 4.98)	4.00 (3.75, 4.65)
**Supplement**			
no Supplement	107 (85%)	93 (85%)	14 (88%)
Supplement	19 (15%)	17 (15%)	2 (12%)

Median (IQR); n (%)

1.1 Supplementary Figures

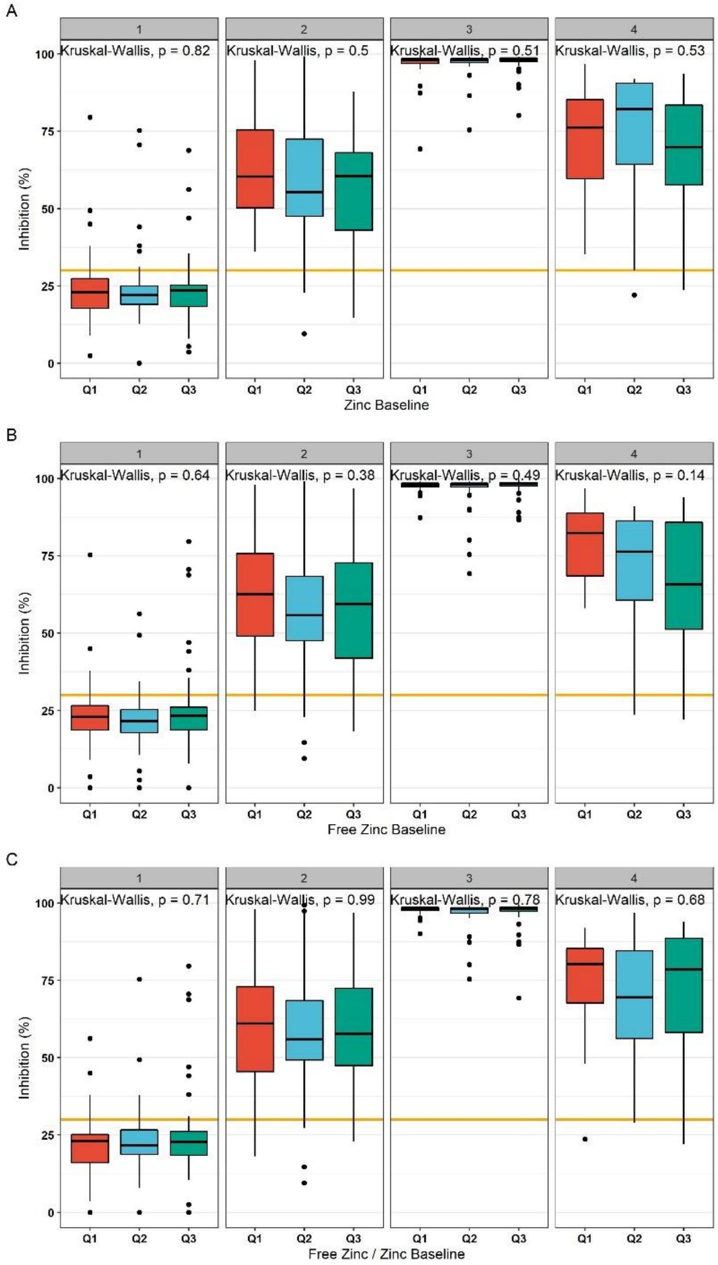

Figure S1. Baseline Zn status in relation to neutralizing potency of antibodies. (**A**) Total serum Zn was categorized into tertiles (Q1 < 764.3 µg/L; Q2 < 852.4 µg/L; Q3 > 852.4 µg/L). No significant difference in inhibition activities were observed. (**B**) Free zinc was divided into tertiles (Q1 <0.51 nM; Q2 < 0.59 nM; Q3 > 0.59 nM), and no significant differences in neutralization ability was detected. (**C**) The free Zn/total serum Zn ratios were classified into tertiles (Q1 < 4.09; Q2 < 4.81; Q3 > 4.81). Again, no differences in inhibition potency of the SARS-CoV-2 antibodies were found across the tertiles. Two-sided Kruskal-Wallis test was used to assess differences.

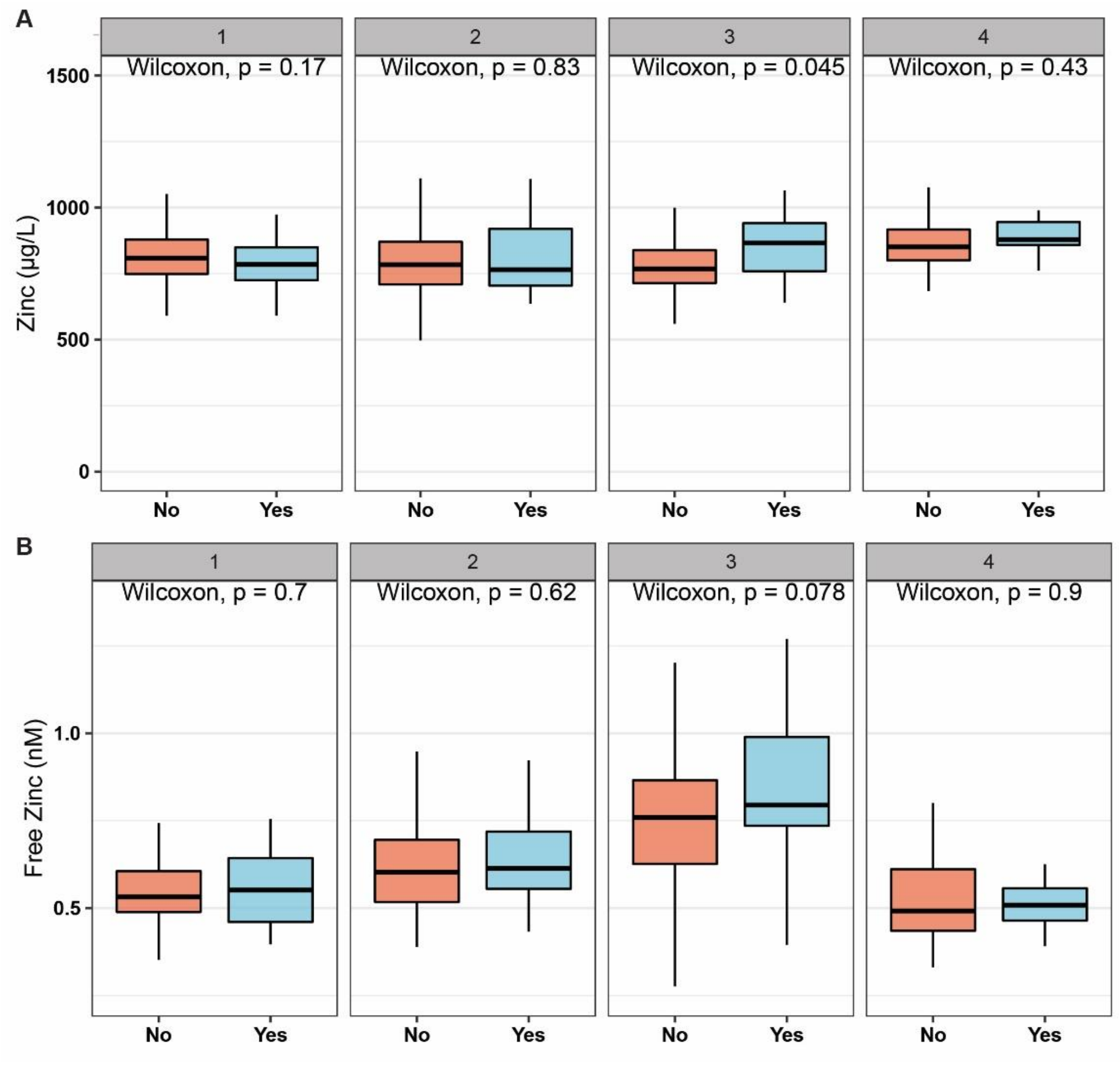

Figure S2. Total and free serum Zn concentrations in relation to Zn supplementation. A subgroup of subjects reported self-administered Zn supplement intake during the study. No significant differences in total serum Zn (**A**) or free Zn concentrations (**B**) were detected by the quantitative analysis of the serum parameters. Pairwise comparisons were conducted by applying the Wilcoxon-Rank-Sum test.

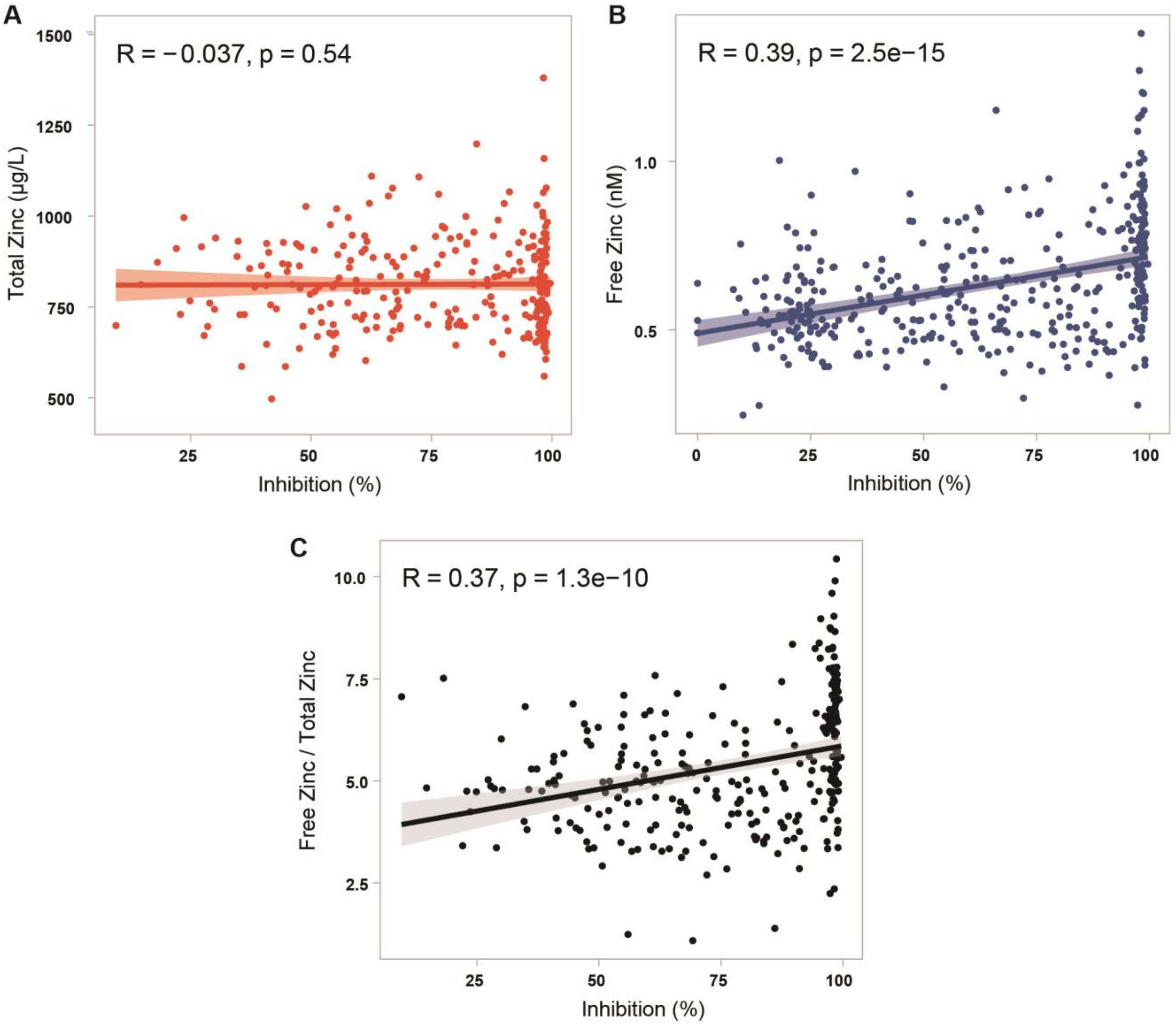

Figure S3. Correlation analysis of the three biomarkers of Zn status with respect to neutralizing antibody concentrations. **(A)** Total serum Zn and inhibition index show no significant correlation (three data points with high Zn concentrations are not shown for reasons of scale). In comparison, the parameters (**B**) free Zn and **(c)** free Zn/total serum Zn ratio display significant correlations with the inhibition index of the antibodies across all samples. Data were analysed by Spearman's rank correlation.
